# Lytic polysaccharide monooxygenases from *Myceliophthora thermophila* C1 differ in substrate preference and reducing agent specificity

**DOI:** 10.1186/s13068-016-0594-y

**Published:** 2016-08-31

**Authors:** Matthias Frommhagen, Martijn J. Koetsier, Adrie H. Westphal, Jaap Visser, Sandra W. A. Hinz, Jean-Paul Vincken, Willem J. H. van Berkel, Mirjam A. Kabel, Harry Gruppen

**Affiliations:** 1 Laboratory of Food Chemistry, Wageningen University, Bornse Weilanden 9, 6708 WG Wageningen, The Netherlands; 2 DuPont Industrial Biosciences, Nieuwe Kanaal 7-S, 6709 PA Wageningen, The Netherlands; 3 Laboratory of Biochemistry, Wageningen University, Stippeneng 4, 6708 WE Wageningen, The Netherlands; 4 Fungal Genetics & Technology Consultancy, P.O. Box 39b, 6700 AJ Wageningen, The Netherlands

**Keywords:** Electron donor, Phenolics, Flavonoids, Lignin, Glucan, Xylan

## Abstract

**Background:**

Lytic polysaccharide monooxgygenases (LPMOs) are known to boost the hydrolytic breakdown of lignocellulosic biomass, especially cellulose, due to their oxidative mechanism. For their activity, LPMOs require an electron donor for reducing the divalent copper cofactor. LPMO activities are mainly investigated with ascorbic acid as a reducing agent, but little is known about the effect of plant-derived reducing agents on LPMOs activity.

**Results:**

Here, we show that three LPMOs from the fungus *Myceliophthora thermophila* C1, *Mt*LPMO9A, *Mt*LPMO9B and *Mt*LPMO9C, differ in their substrate preference, C1-/C4-regioselectivity and reducing agent specificity. *Mt*LPMO9A generated C1- and C4-oxidized, *Mt*LPMO9B C1-oxidized and *Mt*LPMO9C C4-oxidized gluco-oligosaccharides from cellulose. The recently published *Mt*LPMO9A oxidized, next to cellulose, xylan, β-(1 → 3, 1 → 4)-glucan and xyloglucan. In addition, *Mt*LPMO9C oxidized, to a minor extent, xyloglucan and β-(1 → 3, 1 → 4)-glucan from oat spelt at the C4 position. In total, 34 reducing agents, mainly plant-derived flavonoids and lignin-building blocks, were studied for their ability to promote LPMO activity. Reducing agents with a 1,2-benzenediol or 1,2,3-benzenetriol moiety gave the highest release of oxidized and non-oxidized gluco-oligosaccharides from cellulose for all three *Mt*LPMOs. Low activities toward cellulose were observed in the presence of monophenols and sulfur-containing compounds.

**Conclusions:**

Several of the most powerful LPMO reducing agents of this study serve as lignin building blocks or protective flavonoids in plant biomass. Our findings support the hypothesis that LPMOs do not only vary in their C1-/C4-regioselectivity and substrate specificity, but also in their reducing agent specificity. This work strongly supports the idea that the activity of LPMOs toward lignocellulosic biomass does not only depend on the ability to degrade plant polysaccharides like cellulose, but also on their specificity toward plant-derived reducing agents in situ.

**Electronic supplementary material:**

The online version of this article (doi:10.1186/s13068-016-0594-y) contains supplementary material, which is available to authorized users.

## Background

Plant biomass utilization is considered to be a green approach for the production of renewable biofuels and biochemicals. Here, current developments aim at the effective degradation of the plant biomass polysaccharides, mostly embedded in a lignocellulosic complex, into monosaccharides using enzyme cocktails. These commercial enzyme preparations usually originate from fungi such as *Aspergillus* and *Trichoderma* strains. Alternatively, the ascomycete *Myceliophthora thermophila* C1 is used to produce plant polysaccharide-degrading enzymes [1–3].

Lignocellulosic plant biomass is composed of the aromatic heteropolymer lignin and polysaccharides, such as hemicellulose and cellulose. In this research, we focus on the degradation of the highly recalcitrant polysaccharide cellulose. Cellulose is a homogenous polymer consisting of β-(1 → 4)-linked glucosyl chains. The interactions of these glucosyl chains via hydrogen bondings and van der Waal forces lead to the formation of crystalline cellulose regions [4]. These crystalline regions are difficult to access for most of the known hydrolytic cellulases listed in the Carbohydrate-Active enZyme (CAZy, [5]) database. The recently discovered lytic polysaccharide monooxygenases (LPMOs) are able to improve the hydrolytic breakdown of crystalline cellulose regions by their oxidative mechanisms [6, 7]. It has also been shown that certain LPMOs oxidize chitin, hemicellulosic glucan, soluble cellodextrins, xylan or starch [2, 8–12]. LPMOs are classified as “auxiliary activities” (AA) and are divided, based on their sequence similarity, into four CAZy subgroups: AA9, AA10, AA11 and AA13 [5].

The AA9 classified fungal LPMOs exhibit the oxidative cleavage of cellulose-yielding products with either the C1- (lactones) or C4- (ketoaldoses) position oxidized, or mixtures of the two [6, 13–15]. The oxidative cleavage of substrates by LPMOs requires a divalent copper ion in the active site in addition to molecular oxygen and a reducing agent to oxidize one β-(1 → 4)-linkage between the glucosyl residues [15].

Most studies on the activity of LPMOs published so far have been carried out with ascorbic acid as reducing agent [2, 6, 10, 16, 17]. Besides ascorbic acid, other small molecular weight reducing agents have been described to donate electrons to LPMOs, such as hydroquinone, catechin, and gallic acid and the macromolecule lignin [9, 13, 14, 18–20]. Interestingly, it has been shown that reducing agents, especially the plant-derived diphenols, can be regenerated by GMC oxidoreductases [20]. Furthermore, flavocytochrome-dependent cellobiose dehydrogenases (CDHs) are reported to provide LPMOs with electrons and, more recently, light-excited photosynthetic pigments [21–23]. Literature is, however, ambiguous whether the type of reducing agent also influences the LPMO activity. On the one hand, it has been shown that the addition of different reducing agents influences the amount of released oxidized and non-oxidized gluco-oligosaccharides from PASC (phosphoric acid swollen cellulose) incubated with LPMO [19, 20]. On the other hand, the addition of three different reducing agents to PASC incubated with *Pc*GH61D did not affect the amounts of products released [7]. Therefore, it remains unknown whether different LPMOs share the same preference for the same type of reducing agent. In addition, for fungi it has been shown that polysaccharide sources used for fungal growth influence the expression of LPMO encoding genes [3]. Hence, we hypothesize that different LPMOs from *M. thermophila* C1 do not only vary in their C1-/C4-regioselectivity and substrate specificity, but also in their reducing agent specificity [3]. Therefore, we investigated the effect of 34 different reducing agents, in particular plant-derived reducing agents, such as flavonoids or lignin building blocks, on the cellulose-degrading activity of three LPMOs from *M. thermophila* C1. One of these LPMOs is *Mt*LPMO9A, which has been shown to oxidize cellulose at the C1- and C4 position [2]. For the two other LPMOs, *Mt*LPMO9B and *Mt*LPMO9C, we also characterized the C1-/C4-regioselectivity and substrate specificity. Based on their chemical structure, all reducing agents were classified into five groups, partly reflecting their effect on the LPMO activity.

## Results

### Purification of *Mt*LPMO9B and *Mt*LPMO9C

*Mt*LPMO9B and *Mt*LPMO9C were produced in the homologous host *M. thermophila* C1 and purified from the culture broth supernatant in either three (*Mt*LPMO9B) or four (*Mt*LPMO9C) steps. Both final *Mt*LPMO9B and *Mt*LPMO9C preparations showed single bands on SDS-PAGE (Additional file 1) with apparent subunit molecular masses of 32 and 25 kDa, respectively. The purified *Mt*LPMO9B and *Mt*LPMO9C preparations were further analyzed by LC/UV/ESI-MS. The weighted average masses of *Mt*LPMO9B and *Mt*LPMO9C were 32,765 and 24,640 Da, respectively. These values are somewhat higher than the theoretical molecular masses (30.6 and 23.5 kDa, respectively) calculated from the respective amino acid sequences. However, ESI-MS spectra (*m/z* values) of *Mt*LPMO9B and *Mt*LPMO9C showed that both LPMOs were glycosylated by hexoses. In particular, up to 13 and 5 glycosyl units were attached to *Mt*LPMO9B and *Mt*LPMO9C, respectively (Additional file 2).

### The mode of action of *Mt*LPMO9B and *Mt*LPMO9C on amorphous cellulose

**Fig. 1:**
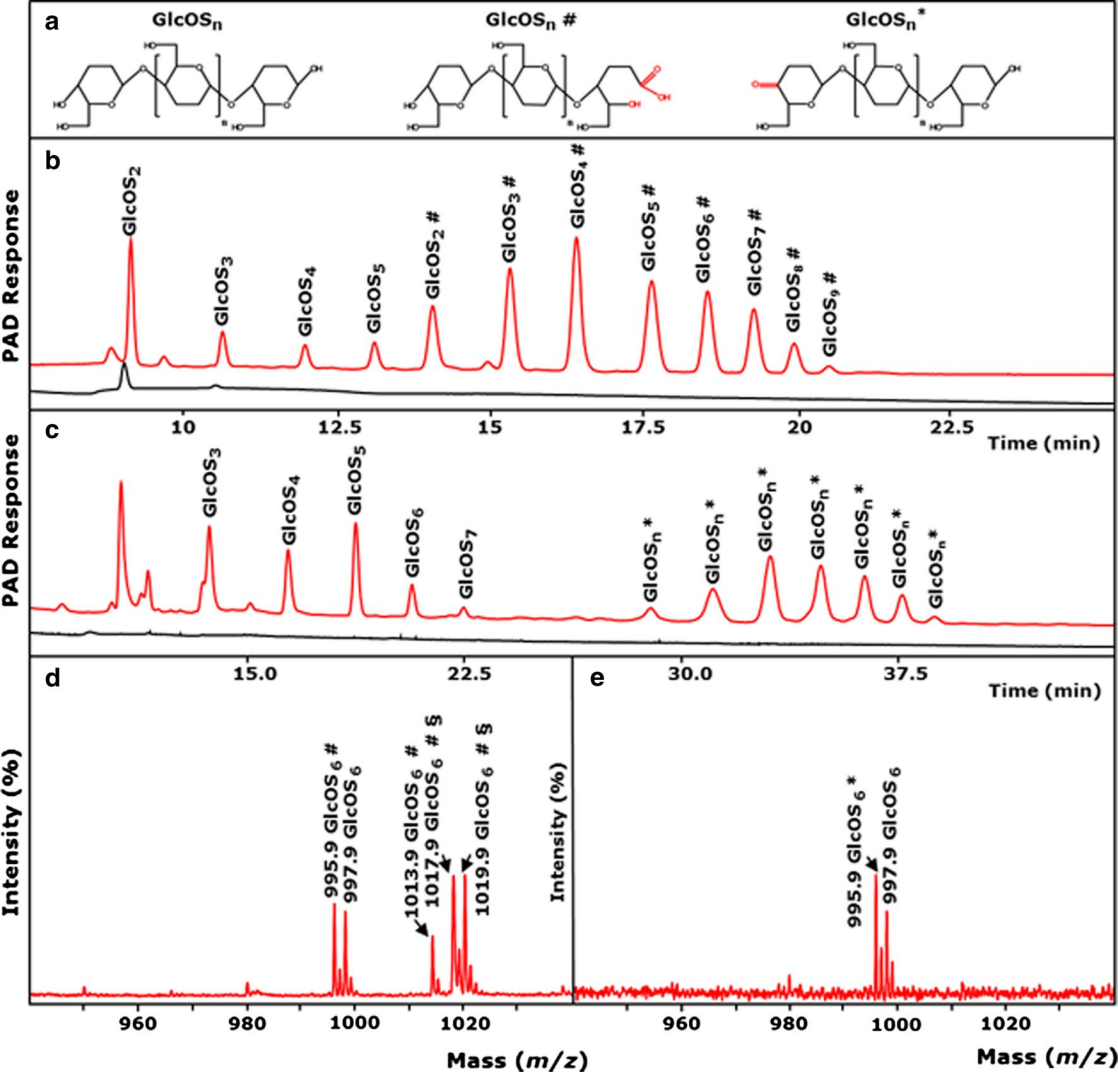
Activity of *Mt*LPMO9B and *Mt*LPMO9C on regenerated amorphous cellulose. **a** Structure and nomenclature of released non-oxidized and C1- and C4-oxidized gluco-oligosaccharides (GlcOS_n_, GlcOS_n_^#^, GlcOS_n_^*^, respectively). HPAEC elution pattern of regenerated amorphous cellulose (RAC; 2 mg × mL^−1^) incubated with b *Mt*LPMO9B (10 mg × g^−1^ substrate) and **c** *Mt*LPMO9C (10 mg × g^−1^ substrate), in the presence (1 mM, *red line*) and absence of ascorbic acid (*black line*). A different gradient was used for the separation of **b** C1- and **c** C4-oxidized gluco-oligosaccharides (See “Methods”). The C4-oxidized gluco-oligosaccharides are known to be unstable under the alkaline conditions present during HPAEC analysis and undergo further derivatization to gem-diols, which are actually annotated as C4-oxidized gluco-oligosaccharides (GlcOS_n_^*^) [34]. MALDI-TOF mass spectrum (*m*/*z* values) of RAC incubated with **d** *Mt*LPMO9B or **e** *Mt*LPMO9C, in the presence of ascorbic acid. **d** Double Li^−^ adducts of C1-oxidized gluco-oligosaccharides are marked with *section symbol*. **d** Oxidation of the C1-carbon atom results in the formation non-oxidized gluco-oligosaccharides (GlcOS_n_) and C1-oxidized gluco-oligosaccharides present as a δ-lactone (−2 Da, marked as GlcOS_n_^#^). Lactones are unstable and convert to aldonic acids by the addition of water, leading to a 16 Da higher mass compared to the non-oxidized gluco-oligosaccharide (+16 Da, marked as GlcOS_n_^#^). Double Li^−^ adducts of C1-oxidized gluco-oligosaccharides are marked with *section symbol* (GlcOS_6_^#^§, 1019.9 and 1017.9 Da, probably due to double oxidation). **e** Oxidation of the C4-carbon atom results in the formation of non-oxidized gluco-oligosaccharides (GlcOS_n_) and C4-oxidized gluco-oligosaccharides present as ketoaldoses (−2 Da, marked as GlcOS_n_^*^). No gem-diols were formed. For more information see “Methods”

The activities of the pure *Mt*LPMO9B and *Mt*LPMO9C enzymes were tested on regenerated amorphous cellulose (RAC), in both the presence and absence of ascorbic acid. Incubation of RAC in the presence and absence of ascorbic acid without LPMO addition did not cause auto-oxidation of RAC, since no non-oxidized or oxidized gluco-oligosaccharides were detected using HPAEC and MALDI-TOF MS. The products released from RAC, after incubation with *Mt*LPMO9B or *Mt*LPMO9C, are shown in Fig. 1. Besides non-oxidized gluco-oligosaccharides (GlcOS_n_), only C1-oxidized gluco-oligosaccharides (GlcOS_n_^#^) were formed upon incubation of RAC with *Mt*LPMO9B in the presence of ascorbic acid (Fig. 1b). In contrast, RAC incubated with *Mt*LPMO9C in the presence of ascorbic acid yielded only C4-oxidized gluco-oligosaccharides (GlcOS_n_^*^), besides non-oxidized gluco-oligosaccharides (GlcOS_n_) (Fig. 1c). For both enzymes, neither oxidized nor non-oxidized gluco-oligosaccharides were released from RAC in the absence of ascorbic acid, which showed that cellulolytic activity was completely absent (Fig. 1b, c). MALDI-TOF MS confirmed the annotation of the HPAEC eluted gluco-oligosaccharides (Fig. 1d, e; Additional file 3a, b). For example, incubation of RAC with *Mt*LPMO9B released non-oxidized cellohexaose with a mass of 997 Da (lithium adduct) and C1-oxidized cellohexaose appeared either as a lactone (995 Da) or as the corresponding aldonic acid (1013 Da) (Fig. 1d). Masses of lactonic acid double substituted with lithium were also detected (1019 and 1017 Da, probably due to double oxidation). In contrast, RAC incubated with *Mt*LPMO9C only formed the C4-oxidized ketoaldose (995 Da) of cellohexaose and non-oxidized cellohexaose (997 Da, Fig. 1e). In summary, *Mt*LPMO9B oxidizes cellulose at the C1- and *Mt*LPMO9C at the C4-position, while the previously characterized *Mt*LPMO9A oxidizes cellulose at the C1- and C4-positon [2].

### Structure-based sequence alignment of *Mt*LPMO9A, *Mt*LPMO9B and *Mt*LPMO9C and structural models of *Mt*LPMO9B and *Mt*LPMO9C

**Fig. 2:**
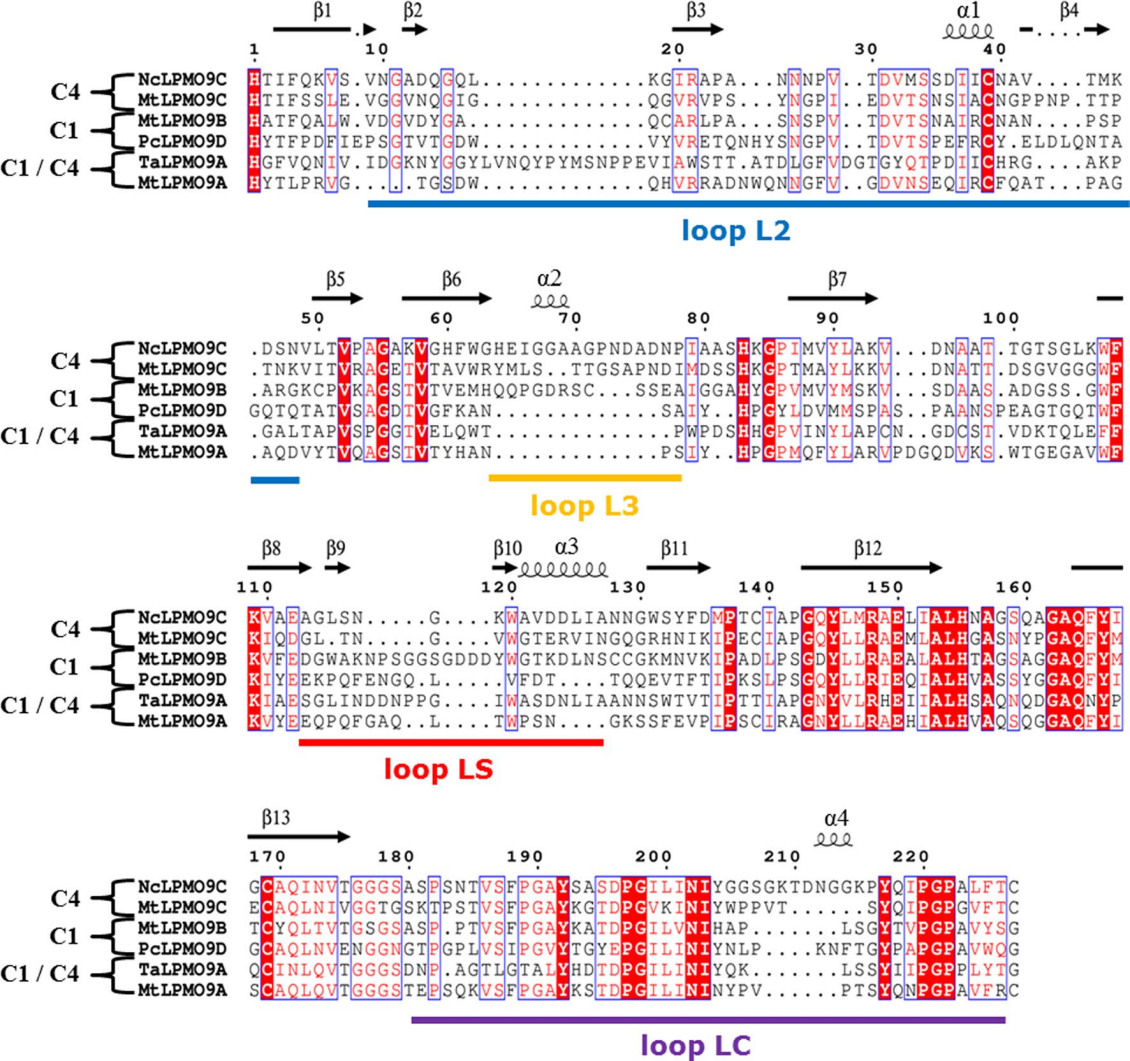
Structure-based sequence alignment of *Mt*LPMO9A, *Mt*LPMO9B and *Mt*LPMO9C. The structure-based sequence alignments of *Mt*LPMO9A, *Mt*LPMO9B and *Mt*LPMO9C were made using three known sequences of a C1-(*Pc*LPMO9D; PDB ID code 4B5Q), C4-(*Nc*LPMO9C; PDB ID code 4D7U) and C1- and C4-(*Ta*LPMO9A; PDB ID code 3ZUD) oxidizing LPMO based on Borisova et al. [10, 14, 24, 25]. The β-strands (*black arrow*) and α-helices (*black helix*) are based on *Nc*LPMO9C and shown above the columns [25]. The *bold colored lines* below the columns highlight the amino acids of the four loop regions L2 (*blue*), L3 (*yellow*), Ls (*red*) and LC (*purple*), which are involved in shaping the substrate-binding site. The highly conserved amino acid residues are presented as *white letters* on a *red background*. Amino acid residues that have comparable chemical and physical properties are presented as *red letters* within *blue frames*. Sequences are presented without the signal sequence and start from the N-terminal histidine (His1). The structure-based sequence alignment was obtained using ESPript [48]

**Fig. 3:**
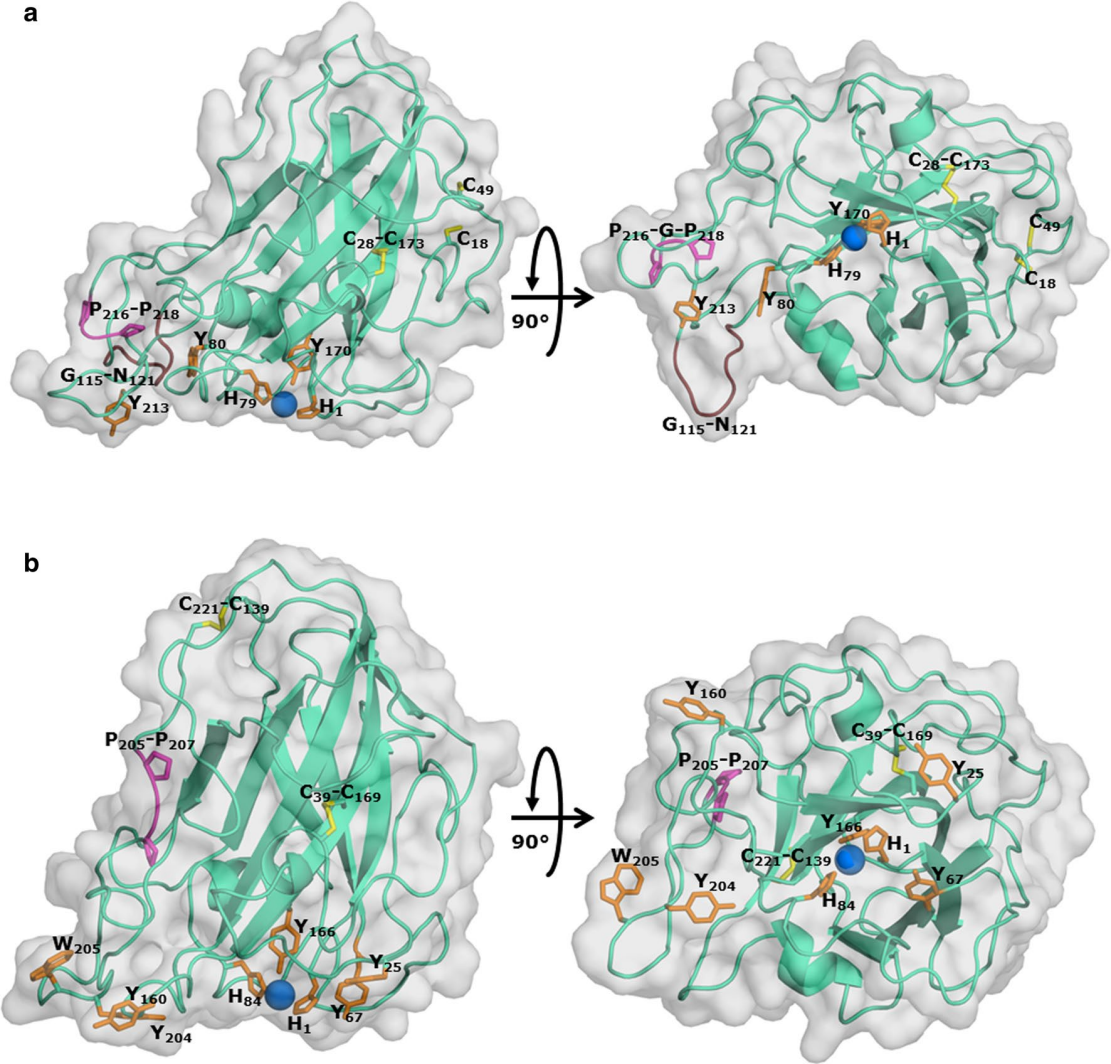
Structural models of *Mt*LPMO9B and *Mt*LPMO9C. The structural model of **a** *Mt*LPMO9B was generated based on the available structure of *Nc*LPMO9C from *Neurospora crassa* [25] (PDB entry: 4D7U). The amino acid sequence identities of *Mt*LPMO9B and *Mt*LPMO9C compared to *Nc*LPMO9C were 41 and 46 %, respectively. The copper ion (*blue*) is coordinated by His1, His 79 and Tyr170 (*orange*). A disulfide bridge is located between Cys28 and Cys178 (*yellow*) and it is likely that the neighboring Cys18 and Cys49 form a second disulfide bridge. The structural model of **b** *Mt*LPMO9C was, like *Mt*LPMO9B, generated based on *Nc*LPMO9C [25] (PDB entry: 4D7U). The copper ion (*blue*) is coordinated by His1, His84 and Tyr166 (orange). *Mt*LPMO9C contains two disulfide bridges between Cys39 and Cys169, and Cys139 and Cys221 (*yellow*). The highly conserved Gly-Pro-Gly triad (*magenta*) of *Mt*LPMO9B and *Mt*LPMO9C is located between Pro216 and Pro218, and Pro205 and Pro207, respectively. *Mt*LPMO9B contains, unlike *Mt*LPMO9C, an additional loop between Gly-115 and Asn121 (*brown*)

Structure-based sequence alignments of *Mt*LPMO9A, *Mt*LPMO9B and *Mt*LPMO9C were constructed, based on the sequences of a C1-(*Pc*LPMO9D; PDB ID code 4B5Q), C4-(*Nc*LPMO9C; PDB ID code 4D7U) and C1- and C4-(*Ta*LPMO9A; PDB ID code 3ZUD) oxidizing LPMO as presented by Borisova et al. (Fig. 2) [10, 14, 24, 25]. The three-dimensional structural models of *Mt*LPMO9B and *Mt*LPMO9C (Fig. 3a, b) were generated based on the available structure of *Nc*LPMO9C from *Neurospora crassa* [25] (Protein Data Bank entry: 4D7U). *Mt*LPMO9B is, unlike *Mt*LPMO9C and the previously published *Mt*LPMO9A [2], linked at the C-terminal end to a carbohydrate-binding module 1 (CBM1), which is not presented in the structural model of *Mt*LPMO9B. The sequences used for the structural-based alignments and models do not include the signal peptides and start from the N-terminal histidine (His1). The overall sequence identity of *Mt*LPMO9A, *Mt*LPMO9B and *Mt*LPMO9C ranges from 41 to 46 %. All three *Mt*LPMOs share the LPMO typical β-sheet core, but differ in their loop regions L2 (10–49), LS (114–128) and LC (176–226) that are involved in shaping the substrate-binding surface (Fig. 2) [2, 25, 26]. Interestingly, *Mt*LPMO9C and partly *Mt*LPMO9B contain an insertion, which forms the L3 (64–78) loop region (Fig. 2). This L3 region is a typical structural characteristic of C4-oxidizing AA9 LPMOs [25–27]. Based on the model, *Mt*LPMO9B contains distal from the coordinated copper sphere an additional loop (Gly115-Asn121), which is not present in *Mt*LPMO9A and *Mt*LPMO9C (Fig. 2a, b). The copper ion in *Mt*LPMO9A, *Mt*LPMO9B and *Mt*LPMO9C is coordinated by His1-His68-Tyr153, His1-His79-Tyr170 and His1-His84-Tyr166, respectively (Figs. 2, 3) [2]. All three *Mt*LPMOs share two putative disulfide bridges, presumably involved in stabilizing the different loops regions such as Cys126-Cys208 (LS–LC) for *Mt*LPMO9A and Cys28-Cys178 (L2–LC) for *Mt*LPMO9B (Fig. 3) [2]. In addition, it is likely that the neighboring Cys18 and Cys49 of *Mt*LPMO9B form a second disulfide bond (L2–L2) (Fig. 3). The putative disulfide bridges of *Mt*LPMO9C between Cys39-Cys169 and Cys139-Cys221 are not expected to be involved in interlinking any of the four described loop regions (Fig. 2). All three *Mt*LPMOs share the presence of several aromatic amino acid residues in the substrate-binding surface, which were formerly used for classifying the AA9s into subgroups (Figs. 2, 3) [2, 28, 29].

### Activity of *Mt*LPMO9B and *Mt*LPMO9C with various soluble and insoluble polysaccharides

Various soluble and insoluble substrates were incubated with purified *Mt*LPMO9B or *Mt*LPMO9C in the presence of ascorbic acid. The overview of the activities observed is presented in Table 1, which, for comparison, includes the activities of the previously published *Mt*LPMO9A [2]. Of all substrates tested, *Mt*LPMO9B showed only oxidative activity toward RAC, releasing C1-oxidized and non-oxidized gluco-oligosaccharides (Fig. 1b). *Mt*LPMO9C oxidized RAC (Fig. 1c) releasing C4-oxidized and non-oxidized gluco-oligosaccharides and showed also activity toward β-(1 → 3, 1 → 4)-glucan from oat spelt or xyloglucan from tamarind seed under the formation of C4-oxidized gluco-oligosaccharides and substituted C4-oxidized gluco-oligosaccharides (Additional files 4, 5, 6). No oxidized gluco-oligosaccharides were released from β-(1 → 3, 1 → 4)-glucan from barley incubated with *Mt*LPMO9B or *Mt*LPMO9C.

**Table 1:** Oxidation of various polysaccharide substrates by *Mt*LPMO9A*, Mt*LPMO9B and *Mt*LPMO9C

Substrate	Occurrence of oxidation (upon addition of 1 mM ascorbic acid)							
	*Mt*LPMO9A^a^		*Mt*LPMO9B		*Mt*LPMO9C		No enzyme	
	GlcOS_n_^# * b^	XOS_n_^# * c^	GlcOS_n_^#^	XOS_n_^#^	GlcOS_n_*	XOS_n_*	GlcOS_n_*	XOS_n_*
Cellulose								
RAC^d^	+	+	+	–	+	–	–	–
Hemicellulose								
Glucan								
Xyloglucan^e^	+	–	–	–	+	–	–	–
β-Glucan barley^e^	+	–	–	–	–	–	–	–
β-Glucan oat spelt^e^	+	–	–	–	+	–	–	–
Xylan								
OSX^f^	–	–	–	–	–	–	–	–
BiWX^f^	–	–	–	–	–	–	–	–
WAX^f^	–	–	–	–	–	–	–	–
Oligosaccharides								
Gluco-oligosaccharides^g^	–	–	–	–	–	–	–	–
Xylo-oligosaccharides^g^	–	–	–	–	–	–	–	–
RAC/Hemicellulose combination								
RAC + BiWX	+	+	+	–	+	–	–	–
RAC + OSX	+	+	+	–	+	–	–	–
RAC + WAX	+	–	+	–	+	–	–	–

Oligosaccharides released and not released refers to + and −, respectively

^a^Data from Frommhagen et al. **[** 2 **]**

^b^Gluco-oligosaccharides oxidized at the C1 (GlcOS_n_^**#**^) or C4 position (GlcOS_n_*)

^c^Xylo-oligosaccharides oxidized at the C1 (XOS_n_^**#**^) or C4 position (XOS_n_*)

^d^Regenerated amorphous cellulose (RAC)

^e^Xyloglucan from tamarind seed, β-(1 → 3, 1 → 4)-glucan from barley and β-(1 → 3, 1 → 4)-glucan from oat spelt

^f^Oat spelt xylan (OSX), birchwood xylan (BiWX), wheat arabinoxylan (WAX)

^g^β-(1 → 4)-linked gluco- and xylo-oligosaccharides, degree of polymerization 2–5

*Mt*LPMO9A has been described to cleave xylan associated with cellulose [2] forming oxidized xylo-oligosaccharides and oxidized gluco-oligosaccharides (Table 1). Hence, *Mt*LPMO9B and *Mt*LPMO9C were also studied for their activity toward RAC–xylan mixtures, in particular, RAC mixed with either birchwood xylan, oat spelt xylan or wheat arabinoxylan, in the presence and absence of ascorbic acid (Table 1). No oxidized xylo-oligosaccharides were released by *Mt*LPMO9B and *Mt*LPMO9C, which discriminates these enzymes from *Mt*LPMO9A.

### Electron donor specificities of *Mt*LPMO9A, *Mt*LPMO9B and *Mt*LPMO9C

**Fig. 4:**
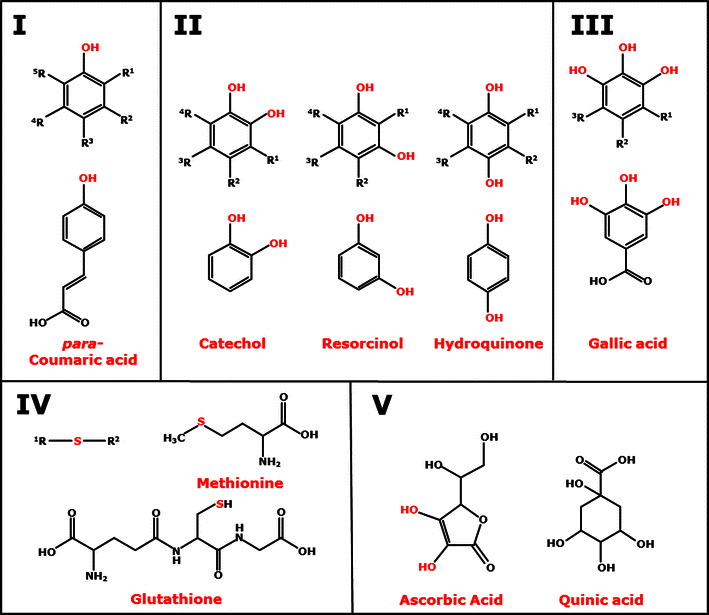
Structural classification of reducing agents into five groups. Group **I**, monophenols. Group **II**, *ortho*-, *meta*- or *para*-isomers of compounds bearing a benzenediol moiety. Group **III**, reducing agents with a 1,2,3-benzenetriol moiety. Group **IV**, sulfur-containing compounds. Group **V**, compounds with neither a phenolic ring nor a sulfur atom

Based on the structural similarities of functional groups, the 34 reducing agents tested were classified into five groups (Fig. 4). Monophenols, like sinapic acid (no. 5), are classified as group I. Group II comprises compounds with a benzenediol moiety, which represent the *ortho*-isomer 1,2-benzenediol, the *meta*-isomer 1,3-benzenediol and the *para*-isomer 1,4-benzenediol (Fig. 4). Reducing agents with a 1,2,3-benzenetriol moiety are classified as group III. Reducing agents of group IV are sulfur-containing compounds, such as glutathione (no. 29) or l-cysteine (no. 30). Reducing agents of group V have neither a phenolic ring nor a sulfur atom.

**Fig. 5:**
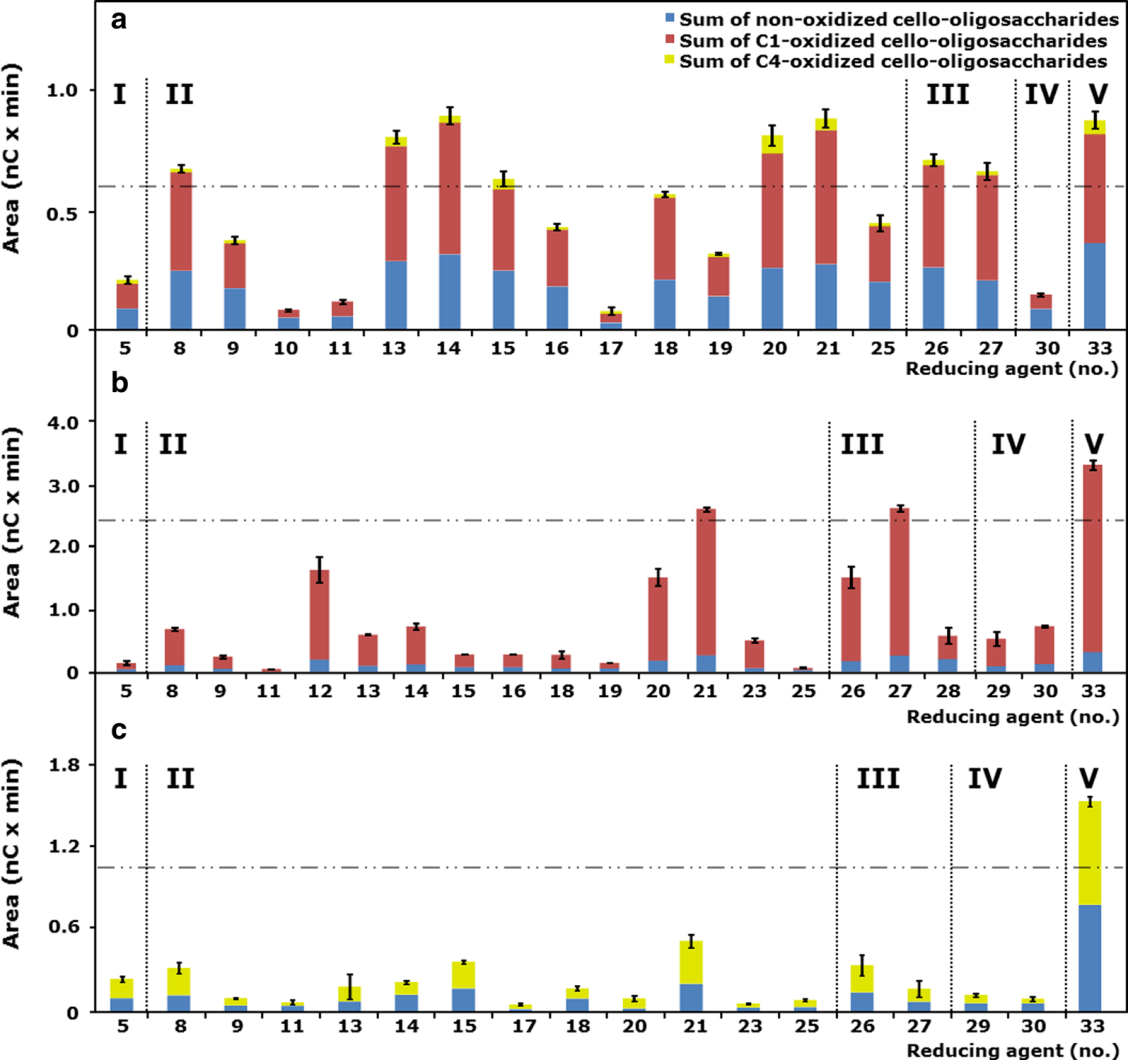
Released oligosaccharides from RAC incubated with three different *Mt*LPMOs. RAC was incubated with **a** *Mt*LPMO9A, **b** *Mt*LPMO9B and **c** *Mt*LPMO9C using different reducing agents. The total sum is shown of integrated peak areas of released C1- (*red*), C4- (*yellow*) oxidized and non-oxidized (*blue*) gluco-oligosaccharides after incubation of *Mt*LPMO9A*, Mt*LPMO9B and *Mt*LPMO9C (2.5, 5 and 2.5 mg × g^−1^ substrate, respectively) with regenerated amorphous cellulose (RAC; 1.5 mg × mL^−1^) based on HPAEC. The reducing agents (1 mM) are numbered (X-axes) and specified in Table 2. *Vertical dotted lines* separate reducing agents of the five structural groups (Fig. 4). The threshold (*horizontal dashed dotted line*) is set to 70 % of the released products from RAC incubated with the *Mt*LPMOs in the presence of ascorbic acid (no. 33) (Table 2). See “Methods” for data analysis. All incubations were performed in duplicates, and the standard deviation is represented by *error bars*, which correspond to one cumulated SD (*error bar* = ± SDtot; with SDtot = √SD_1_^2^ + SD_2_^2^ +…)

An overview of the effect of 34 different reducing agents with corresponding codes (no.) on the amounts of oxidized and non-oxidized gluco-oligosaccharides released from RAC incubated with *Mt*LPMO9A, *Mt*LPMO9B or *Mt*LPMO9C is presented in Table 2. First, we determined from the HPAEC profiles the amounts, expressed as peak areas, of C1-oxidized gluco-oligosaccharides, C4-oxidized gluco-oligosaccharides and non-oxidized gluco-oligosaccharides formed (Fig. 5). Subsequently, these amounts were presented as percentage of the corresponding amounts obtained by the same incubation, but with ascorbic acid (no. 33), which was set to 100 % (Table 2). All 34 reducing agents were also incubated with RAC alone, which did not result in the release of oxidized or non-oxidized gluco-oligosaccharides, confirming the absence of auto-oxidation in the presence of the reducing agents only.

**Table 2:** Overview of electron donor specificity of *Mt*LPMO9A*, Mt*LPMO9B or *Mt*LPMO9C

Group	No.	Reducing agent	*Mt*LPMO9A				*Mt*LPMO9B			*Mt*LPMO9C		
			Activity compared to ascorbic acid %									
			C1-/C4-ox and non-ox	C1-ox	C4-ox	Non-ox	C1-/C4-ox and non-ox	C1-ox	Non-ox	C1-/C4-ox and non-ox	C4-ox	Non-ox
I	1	3-Hydroxy-4-methoxycinnamic acid	0	0	0	0	0	0	0	0	0	0
	2	Homovanillic acid	0	0	0	0	0	0	0	0	0	0
	3	Naringin	0	0	0	0	0	0	0	0	0	0
	4	*p*-coumaric acid	0	0	0	0	0	0	0	0	0	0
	5	*Sinapic acid*	23	23	31	24	4	3	14	15	12	18
	6	Syringic acid	0	0	0	0	0	0	0	0	0	0
	7	Vanillic acid	0	0	0	0	0	0	0	0	0	0
II	8	*(−)-Epicatechin*	77	68	90	27	21	19	34	21	15	26
	9	*(+)-Catechin*	43	47	41	24	7	6	17	6	5	7
	10	3,4-Dihydroxybenzaldehyde	9	13	7	0	0	0	0	0	0	0
	11	*3,4-Dihydroxybenzoic acid*	13	15	13	0	1	1	2	4	5	3
	12	3,4-Dihydroxycinnamic acid	0	0	0	0	49	48	63	0	0	0
	13	*3,4-Dihydroxyphenylacetic acid*	92	79	105	68	18	17	31	12	9	14
	14	*3-Methylcatechol*	102	87	121	49	22	20	38	14	15	12
	15	*4-Chlorocatechol*	72	68	75	74	8	7	24	24	21	26
	16	Caffeic acid	49	49	52	20	8	7	26	0	0	0
	17	Carminic acid	8	7	8	21	0	0	0	3	2	4
	18	*Catechol*	65	57	75	26	8	7	18	11	12	10
	19	Chlorogenic acid	36	38	36	25	4	3	18	0	0	0
	20	*Dopamine hydrochloride*	93	71	105	132	46	45	57	6	2	10
	21	*l* *-3,4-Dihydroxyphenylalanine*	101	75	123	86	79	78	84	33	26	41
	22	Quercetin	0	0	0	0	0	0	0	0	0	0
	23	Taxifolin	0	0	0	0	15	15	20	4	3	4
	24	Resorcinol	0	0	0	0	0	0	0	0	0	0
	25	*Hydroquinone*	51	55	51	25	2	1	10	5	4	7
III	26	*Gallic acid*	81	72	94	38	46	45	54	22	18	26
	27	*(−)-Epigallocatechin-gallate*	76	56	97	30	79	79	83	11	9	12
	28	Tannic acid	0	0	0	0	18	13	66	0	0	0
IV	29	Reduced glutathione	0	0	0	0	16	15	30	8	7	8
	30	*l* *-cysteine*	16	23	13	0	22	20	40	6	7	4
	31	Allyl iso-thiocyanate	0	0	0	0	0	0	0	0	0	0
	32	d-methionine	0	0	0	0	0	0	0	0	0	0
V	33	*Ascorbic acid*	100	100	100	100	100	100	100	100	100	100
		Peak area (nC x min)^a^	(0.897)	(0.460)	(0.058)	(0.360)	(3.273)	(2.962)	(0.311)	(1.542)	(0.760)	(0.781)
	34	d-quinic acid	0	0	0	0	0	0	0	0	0	0

The total sum of integrated peak areas of released oxidized (C1-ox, C4-ox) and non-oxidized (non-ox) and both (ox and non-ox) gluco-oligosaccharides from RAC, incubated with either *Mt*LPMO9A*, Mt*LPMO9B or *Mt*LPMO9C in the presence of ascorbic acid, was taken as a reference and equals to 100 % of the LPMO activity. The numbers correspond to the relative activity of *Mt*LPMO9A*, Mt*LPMO9B or *Mt*LPMO9C compared to ascorbic acid (total sum integrated peak areas of released oxidized and non-oxidized gluco-oligosaccharides of a reducing agent compared to ascorbic acid (areas from Fig. 5). Auto-oxidation of RAC was absent for all reducing agents tested based on the absence of oxidized or non-oxidized gluco-oligosaccharides. Reducing agents that can donate electrons to all three LPMOs are highlighted in italics. See “Methods” for details about the activity assays

^a^Integrated peak areas of released C1-, C4-oxidized and non-oxidized gluco-oligosaccharides after incubation of *Mt*LPMO9A*, Mt*LPMO9B and *Mt*LPMO9C with RAC in the presence of 1 mM ascorbic acid based on HPAEC (Fig. 5)

Fourteen reducing agents (Table 2, italics), including ascorbic acid, were able to donate electrons to all three *Mt*LPMOs enabling the oxidative cleavage of RAC. Nevertheless, the LPMO activity, based on the amounts of oxidized gluco-oligosaccharides formed, varied per reducing agent (Fig. 5; Table 2). For all three *Mt*LPMOs, ascorbic acid was one of the best electron donors (Table 2). Besides ascorbic acid, compounds bearing a 1,2-benzenediol moiety [3-methylcatechol (no. 14), 3,4-dihydroxyphenylalanine (no. 21)] or 1,2,3-benzenetriol moiety [gallic acid (no. 26), epigallocatechin-gallate (no. 27)] gave the highest formation of oxidized and non-oxidized gluco-oligosaccharides. Interestingly, dopamine (no. 21) turned out to be one of the best electron donors for *Mt*LPMO9A (93 % of the activity compared to ascorbic acid), but was a less efficient electron donor for *Mt*LPMO9B and *Mt*LPMO9C (46 and 6 % compared to ascorbic acid, respectively). Some compounds bearing a 1,2-benzenediol moiety, for example quercetin or taxifolin (no. 23), and the benzenetriol tannic acid (no. 28) did not donate electrons to all three *Mt*LPMOs. Out of seven monophenols, only sinapic acid (no. 5) acted as electron donor for all three *Mt*LPMOs, but less efficient (4–23 %) compared to ascorbic acid (no. 33). A poor electron-donating capacity was also found for sulfur-containing compounds, such as reduced glutathione (no. 29) and l-cysteine (no. 30) (Table 2). Both thiol compounds have already been described as LPMO electron donors in literature [7, 30]. In summary, the presence of different reducing agents strongly influences the release of oxidized and non-oxidized gluco-oligosaccharides from RAC for all three *Mt*LPMOs. Based on these findings, we conclude that *Mt*LPMO9A, *Mt*LPMO9B and *Mt*LPMO9C differ in their specificity toward reducing agents.

## Discussion

The recently discovered LPMOs play a crucial role in the enzymatic degradation of lignocellulosic plant biomass. Here, we purified the C1-oxidizing *Mt*LPMO9B and C4-oxidizing *Mt*LPMO9C, two novel LPMOs from the filamentous fungus *M. thermophila* C1, and compared their catalytic properties with those of the C1- and C4-oxidizing *Mt*LPMO9A [2].

### Protein mass

The difference in molecular mass between *Mt*LPMO9B and *Mt*LPMO9C results from the CBM1 linked to *Mt*LPMO9B. This CBM1 has a predicted mass, calculated from the amino acid sequence of 6811 Da. The weighted average mass of the purified *Mt*LPMO9B linked to CBM1 and *Mt*LPMO9C enzymes (32.765 and 24.640 Da, respectively) differ slightly from the predicted masses, calculated from the amino acid sequences (30.633 and 23.449 Da, respectively). Both *Mt*LPMO9B and *Mt*LPMO9C contain multiple glycosylations that were determined as hexoses based on LC/ESI-MS (Additional file 2). It is known that LPMOs can show glycosylation in the substrate-binding site, though the glycosylation could also appear at the serine/threonine containing linker between the carbohydrate-binding module (CBM), the C-terminal end of the LPMO or at the binding site of the CBM [29, 31, 32].

### Activity on cellulose

The total amount of released oxidized and non-oxidized gluco-oligosaccharides from RAC incubated with *Mt*LPMO9B is approximately three times higher (based on total AUC) compared to the released products from RAC by the *Mt*LPMO9A using ascorbic acid as a reducing agent (Fig. 5). We suggest that the CBM1 attached to *Mt*LPMO9B has a strong affinity to cellulose and supports the activity of *Mt*LPMO9B through the positioning of the *Mt*LPMO9B to the cellulose. Our finding is supported by previous studies which showed that LPMOs linked to a CBM release more oxidized gluco-oligosaccharides from cellulose compared to LPMOs without a CBM [17, 33]. In addition, it has been hypothesized that glycosylation could affect the binding to cellulose, altering the enzyme activity [29]. So far, it has only been demonstrated for CBMs that the glycosylation of proteins with mannose in the planar face can increase the substrate-binding strength toward cellulose and not yet for LPMOs [29, 32]. However, it has been reported that the removal of a CBM1 linked to *Nc*LPMO9C did not show any effect on the degradation rate of amorphous cellulose [25]. Still, a direct comparison of all three LPMOs regarding their activity on cellulose would require the absolute quantification of the released C1- and C4-oxidized gluco-oligosaccharides, which is so far not possible due to the lack of standards. Especially, the quantification of C4-oxidized products has its limits due to the instability of these compounds under alkaline conditions during HPAEC analysis [34]. The release of high amounts of non-oxidized gluco-oligosaccharides observed in the HPAEC spectra is likely to derive, to a certain extent, from the decomposition of these labile C4-oxidized gluco-oligosaccharides into non-oxidized compounds (Fig. 1) [34].

### Substrate specificity and C1-/C4-regioselectivity

*Mt*LPMO9A, *Mt*LPMO9B and *Mt*LPMO9C were compared regarding their substrate specificity and C1-/C4-regioselectivity of oxidation using a wide range of polysaccharides (Table 1). The activity of *Mt*LPMO9A toward various polysaccharides has already been investigated previously, showing that *Mt*LPMO9A oxidizes cellulose, besides β-(1 → 3, 1 → 4)-glucan from oat spelt or barley and xyloglucan from tamarind seed, at the C1 and C4 position. In addition, *Mt*LPMO9A also oxidized xylan in the presence of cellulose [2]. *Mt*LPMO9B oxidized RAC at the C1 position. The presence of the CBM1 linked to *Mt*LPMO9B is not expected to have an impact on the substrate specificity of *Mt*LPMO9B. It has been shown that the removal of a CBM1 linked to *Nc*LPMO9C, which has a broad substrate specificity, does not affect the ability to oxidize different polysaccharides such as cellulose, cellopentaose or xyloglucan [25]. Besides that, *Mt*LPMO9B shows a typical insertion, which forms the L3 loop region in C4-oxidizing AA9 LPMOs. Compared to the solely C4-oxidizing *Nc*LPMO9C, *Mt*LPMO9B shares within this L3 loop only a low sequence identity [10, 25]. The L3 loop is known to be an extension of the surface-binding site and involved in the binding of soluble polysaccharides such as xyloglucan and cello-oligosaccharides [25–27]. *Mt*LPMO9C also contains the L3 loop and is, like *Nc*LPMO9C, a C4-oxidizing AA9 LPMO (Fig. 2). In addition to cellulose, *Mt*LPMO9C oxidizes to a lower extent hemicellulose like β-(1 → 3, 1 → 4)-glucan from oat spelt and xyloglucan from tamarind seed at the C4 position (Additional files 4, 5, 6). The previously published *Nc*LPMO9D (PDB id: 4EIR) from *N. crassa* shares the highest sequence identity (83 %) with *Mt*LPMO9C, but unlike *Mt*LPMO9C it has not been tested if *Nc*LPMO9D is active on hemicellulose such as (1 → 3, 1 → 4)-glucan [15, 29].

### Reducing agent specificity

We show here that three LPMOs from the same organism differ in their reducing agent specificity. Importantly, all three LPMOs are able to utilize various natural phenolic compounds as reducing agents. Most of these reducing agents are present in plants, either free or as lignin building blocks, such as sinapic acid (no. 5), or as flavonoids such as catechin (no. 9) and dopamine (no. 20). This finding is of high relevance as these reducing agents can act as intrinsic electron donors in plant biomass biorefinery [35].

For all three *Mt*LPMOs, phenolic compounds with 1,2-benzenediol and 1,2,3-benzenetriol moieties yielded the highest release of oxidized and non-oxidized gluco-oligosaccharides from cellulose compared to monophenols or sulfur-containing compounds (Fig. 5; Table 2). This observation may be related to the fact that the donation of an electron by one hydroxyl group leads to a dislocation of the π-electron sextet, which is energetically unfavorable. It has been shown that monophenols have a higher oxidation potential compared to compounds with 1,2-benzenediol and 1,2,3-benzenetriol moieties [20]. This high oxidation potential of monophenols hinders the reduction of the active site copper of LPMOs. In contrast to monophenols, phenolic compounds with 1,2-benzenediol and 1,2,3-benzenetriol moieties can stabilize the dislocation of π-electrons by their additional(s) hydroxyl groups due to the resonance effect [36]. Compared to monophenols, these compounds have a low reduction potential and are able to reduce the active site copper of the LPMOs [20]. In addition, the ability of such compounds to donate electrons is also influenced by other electron-donating or electron-withdrawing substituents attached to the aromatic ring [37, 38].

**Fig. 6:**
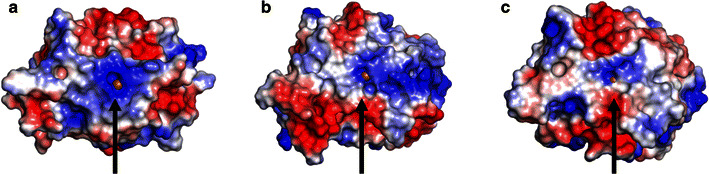
Cartoons of the surface charge distribution of the structural models of **a** *Mt*LPMO9A, **b** *Mt*LPMO9B and **c** *Mt*LPMO9C. Protein orientation: the flat substrate-binding site (Fig. 3) is located to the front of all three LPMOs and the copper ion is indicated by the *black arrow*. Recent NMR studies revealed a direct interaction of the reductant CDH with a narrow surface patch in the vicinity of the copper ion [26]. *Mt*LPMO9A is strongly positively charged in the vicinity of the copper ion compared to *Mt*LPMO9B and *Mt*LPMO9C based on the surface charge distribution (pH 5.0). The scaling from the negative and positive electrostatic potential regions are −5 for *blue* and +5 for the *red* regions. The electrostatic map was obtained from APBS plugin from PyMOL

Remarkably, the number of reducing agents that gave an activity of 70 % or higher of the LPMO activity obtained with ascorbic acid differed between the three LPMOs (Fig. 5; Table 2). For *Mt*LPMO9A this number was eight (compounds with a benzenediol and benzenetriol moiety), but for *Mt*LPMO9B this number was only two (Fig. 5; Table 2). *Mt*LPMO9C oxidizes RAC in the presence of several reducing agents, but, in comparison to *Mt*LPMO9A and *Mt*LPMO9B, none of the reducing agents tested gave an activity of 70 % or higher of the *Mt*LPMO9C-activity obtained with ascorbic acid. Remarkably, we also found reducing agents with a 1,2-benzenediol moiety that are not able to reduce the active site copper of all three *Mt*LPMOs tested (Table 2). These findings cannot be explained by the oxidation potential of the reducing agents alone. Another explanation for the reducing agent preferences among the three LPMOs may result from differences in the protein structure. It has been formerly hypothesized that the binding site of electron-donating proteins, such as CDHs, is located in the surface patch centered around the Pro-Gly-Pro triad, which is highly conserved within the LPMO family [21, 29]. However, recent analysis based on CDH docking studies and NMR revealed a direct interaction of the CDH with the LPMO involving a narrow surface patch around the His1, Ala80, His83 and His155 of *Nc*LPMO9C [21, 26]. Indeed, the surface charge distribution obtained from the homology models differs widely among the three *Mt*LPMOs, including shape and charge in the vicinity of the above described surface patch (Fig. 6; Additional file 7). *Mt*LPMO9A is strongly positively charged in the vicinity of the copper ion compared to *Mt*LPMO9B and *Mt*LPMO9C (pH 5.0). The charge differences in the vicinity of the copper ion might contribute to the different electron donor specificities of the *Mt*LPMOs.

The incubations performed in this work have been conducted at a single time point (24 h) and at one pH (5.0). This pH plays an important role for the LPMO application, due to the fact that cellulose cleaving cocktails produced by *M. thermophila* C1 have their pH optimum around pH 5.0. It can be expected that this pH does not represent the optimal condition for each reducing agent tested, since redox potentials of reducing agents are pH dependent [37]. Furthermore, the product release determined at a single time point (24 h) does not give information about the progress of the LPMO reaction. The time point of 24 h could lie in the initial rate period or already at the end point of the LPMO reaction, which highly depends on the reducing agents present during the reaction [17, 33]. We do not expect a release of non-oxidized or oxidized gluco-oligosaccharides after 24 h if no products have been released from RAC incubated with LPMOs before that time point (Table 2). Finally, the conditions chosen for all three LPMOs have been the same, which allows the comparison of the LPMO activity in the presence of different reducing agents and the chosen conditions can be considered as industrially relevant.

## Conclusions

Our findings support the hypothesis that LPMOs do not only vary in their C1-/C4-regioselectivity and substrate specificity, but also in their reducing agent specificity. The mode of action of LPMOs is usually investigated in the presence of ascorbic acid. Here, we found that several reducing agents can donate electrons to the LPMOs with a similar efficiency as the commonly used ascorbic acid. Our findings are of high interest for industrial applications as most of these reducing agents are present in plant biomass and can act as intrinsic mediators in biorefinery processes.

## Methods

### Enzyme expression, production and purification

The purification and activity of *Mt*LPMO9A has been previously described [2]. The homologous expression of *Mt*LPMO9B  (UniProt: KX772410) and *Mt*LPMO9C (UniProt: KX772411) was performed using a low protease/low (hemi-) cellulose producing *Myceliophthora thermophila* C1 strain, which has been described elsewhere [39, 40]. The crude enzyme preparations obtained from the fermentation broth were dialyzed against 10 mM potassium phosphate buffer (pH 7.0). *Mt*LPMO9B and *Mt*LPMO9C were purified from the corresponding dialyzed enzyme preparations using an ÄKTA-Explorer preparative chromatography system (GE Healthcare, Uppsala, Sweden).

*Mt*LPMO9B was purified in three subsequent chromatographic steps. For the first anion-exchange step, the *Mt*LPMO9B-containing enzyme preparation was loaded on a Source 30Q column (50 ml, GE Healthcare). A 20 mM potassium phosphate buffer (pH 7.8) was used to pre-equilibrate the column. Elution was performed using a linear gradient from 0 to 1 M NaCl in 20 mM potassium phosphate buffer (pH 7.8) at a flow rate of 10 mL × min^−1^ and monitored at 220 and 280 nm. All fractions were collected and immediately stored on ice. Peak fractions were, based on UV (280 nm), pooled and concentrated by ultrafiltration (Amicon Ultra, molecular mass cut-off of 3 kDa, Merck Millipore, Cork, Ireland) at 4 °C. The concentrated pools were analyzed by SDS-PAGE to determine the *Mt*LPMO9B-containing pool (expected molecular mass 30.6 kDa). After the first purification step, cation exchange chromatography purification was applied. The *Mt*LPMO9B containing pool was subjected to a Source 30S column (50 mL, GE Healthcare) for further purification (second step). A 20 mM sodium acetate buffer (pH 5.0) was used to pre-equilibrate the column. The elution was performed by using a linear gradient from 0 to 1 M NaCl in 20 mM sodium acetate (pH 5.0) at a flow rate of 5 mL × min^−1^. Elution was monitored at 220 and 280 nm. Fractions obtained (10 mL) were immediately stored on ice. Peak fractions were pooled, concentrated and analyzed by SDS-PAGE as described above. In a third purification step, the *Mt*LPMO9B-containing fraction was bound to a Source 30S column (50 mL, GE Healthcare) using a 10 mM sodium acetate buffer (pH 5.0) after pre-equilibration. After protein application, the column was washed with 20 column volumes of starting buffer. Elution was performed using a linear gradient from 0 to 1 M KCl in 20 mM sodium acetate (pH 5.0) at a flow rate of 5 mL × min^−1^ and monitored at 220 and 280 nm. Fractions (3 mL) were immediately stored on ice. Peak fractions were pooled, concentrated and analyzed by SDS-PAGE as described above.

*Mt*LPMO9C was purified in four subsequent chromatographic steps. As a first size exclusion chromatography (SEC) purification step, the *Mt*LPMO9C-rich enzyme preparation (40 mg × mL^−1^) was loaded onto a self-packed Superdex TM-75 column (100 × 3 cm internal diameter, GE Healthcare) and eluted at 5 mL × min^−1^ with a 10 mM potassium phosphate buffer (pH 7.0). Fractions (5 mL) were immediately stored on ice. Peak fractions were, based on UV (280 nm), pooled and concentrated by ultrafiltration as described above. The concentrated pools were analyzed by SDS-PAGE to determine the *Mt*LPMO9C-containing pool (expected molecular mass 23.5 kDa). For the second SEC purification step, the *Mt*LPMO9C-containing pool was loaded again on the Superdex TM-75 column under the same conditions. Fractions (5 mL) were immediately stored on ice. Peak fractions were pooled, concentrated and analyzed by SDS-PAGE to determine the *Mt*LPMO9C-containing pool as described above. The *Mt*LPMO9C-containing pool was dialyzed against a 20 mM Tris–HCl buffer (pH 8.4) using ultrafiltration (Amicon Ultra, molecular mass cutoff of 3 kDa). The dialyzed *Mt*LPMO9C fraction was, for the third purification step, subjected to a Resource Q column (30 × 16 mm internal diameter, GE Healthcare), which was pre-equilibrated in 20 mM Tris–HCl buffer (pH 8.4) (third step). Elution was performed with a linear gradient from 0 to 1 M NaCl in 20 mM Tris–HCl (pH 8.4) over 20 column volumes at 6 mL × min^−1^. Fractions (3 mL) were immediately stored on ice. Peak fractions were pooled, concentrated and analyzed by SDS-PAGE to determine the *Mt*LPMO9C-containing pool as described above. The *Mt*LPMO9C-containing fraction was dialyzed against a 20 mM potassium phosphate buffer (pH 7.0) using ultrafiltration (Amicon Ultra, molecular mass cut-off of 3 kDa) and subjected to a Resource Q column (30 × 16 mm internal diameter, GE Healthcare). The column was equilibrated using a 20 mM potassium phosphate buffer (pH 7.0) and elution was performed using a linear gradient over 20 column volumes at 6 mL × min^−1^. Fractions (3 mL) were immediately stored on ice. Peak fractions were pooled, concentrated and analyzed by SDS-PAGE as described above.

### Protein analysis

The protein content of *Mt*LPMO9B and *Mt*LPMO9C was determined as described previously using a BCA Protein Assay Kit [2]. Furthermore, the purity of the enzymes was analyzed by sodium dodecyl sulfate polyacrylamide gel electrophoresis (SDS-PAGE) as described before [2]. Pure *Mt*LPMO9B and *Mt*LPMO9C fractions were analyzed by LC–mass spectrometry confirming the presence of the two LPMOs by ‘The Scripps Research Institute’ (San Diego, CA, USA).

### LC/ESI-MS

Purified *Mt*LPMO9B and *Mt*LPMO9C preparations (2.5 mg mL^−1^ in 0.1 % (v/v) trifluoroacetic acid) were analyzed using a liquid chromatography/electron spray ionization-mass spectrometry (LC/ESI-MS) as described previously [2].

### Reducing agents

Reducing agents were supplied by Sigma-Aldrich (Steinheim, Germany), unless stated otherwise. Taxifolin was purchased from Extrasynthese (Genay, France), catechol hydrate and chlorogenic acid from Thermo Fisher Scientific (Waltham, MA USA), tannic acid from BDH Chemical Ltd. (Poole, England) and naringin from Fluka Chemie (Buches, Switzerland).

### Carbohydrates

OSX, BiWX, Avicel PH-101, xylo-oligosaccharides (DP1-5) and β-(1 → 4)-linked gluco-oligosaccharides (DP1-5) were supplied by Sigma-Aldrich. WAX and β-(1 → 3, 1 → 4)-glucan from barley and oat spelt (both medium viscosity) were purchased from Megazyme (Bray, Ireland). Xyloglucan from tamarind seed was obtained from Dainippon Sumitomo Pharma (Osaka, Japan). Regenerated amorphous cellulose (RAC) was prepared from Avicel PH-101 as described [2, 41]. Gluconic acid was purchased from Sigma-Aldrich and cellobionic acid ammonium salt from Toronto Research Chemicals (Toronto, Ontario, Canada).

### *Mt*LPMO9A, *Mt*LPMO9B and *Mt*LPMO9C activity assays

Substrates (see figure captions) were dissolved in 50 mM ammonium acetate buffer (pH 5.0) to a concentration of 1–2 mg × mL^−1^, with or without addition of reducing agents listed in Table 1 (final concentration of 1 mM). *Mt*LPMO9A, *Mt*LPMO9B or *Mt*LPMO9C was added (2.5–10.0 µg of protein mg^−1^ substrate, for details see figure captions) and incubated for 24 h at 50 °C in a head-over-tail Stuart rotator in portions of 1 mL total volume (Bibby Scientific, Stone, UK) at 20 rpm. Supernatants of all incubations with and without reducing agent in the presence of LPMOs and of substrates incubated with and without reducing agents in the absence of LPMOs were analyzed by HPAEC and MALDI-TOF MS. The enzyme reactions were stopped by storing samples at −24 °C. All following sample treatments were performed on ice.

### Structural modeling

The structural model of *Mt*LPMO9B was made using the available structure of *Nc*LPMO9C from *Neurospora crassa* [25] (Protein Data Bank entry: 4D7U) as a template, which scored highest in the BLAST search of *Mt*LPMO9B against the Protein Data Bank (41 % amino acid identity). *Mt*LPMO9C was, like *Mt*LPMO9B, generated based on *Nc*LPMO9C [25] (PDB entry: 4D7U, 46 % amino acid identity). All models were created using Modeller version 9.14 [42]. Multiple comparative models were generated, after which the model with the lowest corresponding DOPE score [43] was selected for image generation using Pymol (Pymol, The PyMOL Molecular Graphics System, Version 1.5.0.4 Schrödinger, New York, NY, USA). The following settings were applied to model the surface charge distribution of the LPMOs: The protonation states of the titratable groups at pH 5.0 of *Mt*LPMO9A, *Mt*LPMO9B and *Mt*LPMO9C, respectively, were calculated using H^++^-server with default settings [44–47]. The server pdb-ouput files were used to generate a surface image colored by charge (range between −5 and 5) using the Pymol APBS-tool (Version 1.4r1 L, Schrödinger).

### Hpaec

Enzyme digests were analyzed by high-performance anion-exchange chromatography (HPAEC) with pulsed amperometric detection (PAD) using an HPAEC system (ICS-5000, Dionex, Sunnyvale, CA, USA) as described previously [2]. The temperature of the autosampler was set to 6 °C. For the analysis of C4-oxidized gluco-oligosaccharides released by *Mt*LPMO9C, a longer gradient was used. The gradient elution program was as follows: 0–45 min, linear gradient 0–250 mM NaOAc; 45–52 min isocratic gradient 400–1000 mM NaOAc. This was followed by equilibration (13 min) of the column with the starting conditions. The assignment of the C1- and C4-oxidized gluco-oligosaccharides using HPAEC is based on previous publications [2, 8, 15, 17], while gluconic and cellobionic acids were assigned by available standards (see “Methods”). All incubations were performed in duplicate. Areas were analyzed to determine the effect of the reducing agents on the release of oxidized and non-oxidized gluco-oligosaccharides from RAC incubated with *Mt*LPMO9A, *Mt*LPMO9B or *Mt*LPMO9C. Standard deviations are represented (Fig. 5) by error bars, which correspond to one cumulated SD (error bar = ± SDtot; with SDtot = √SD12 + SD22 +…).

### MALDI-TOF MS

The analysis of substrates incubated with either *Mt*LPMO9B or *Mt*LPMO9C was performed using matrix-assisted laser desorption ionization-time of flight mass spectrometry (MALDI-TOF MS, Bruker Daltonics) as described previously [2]. Masses of lithium-adducted C1- or C4-oxidized gluco-oligosaccharides for RAC incubated with *Mt*LPMO9B or *Mt*LPMO9C, respectively, were determined and assigned as described previously [2].

### Additional files

**Additional file 1: Figure S1.** SDS-PAGE of *Mt*LPMO9B and *Mt*LPMO9C fractions during enzyme purification. *Mt*LPMO9B and *Mt*LPMO9C were purified by multiple chromatographic steps from the crude enzyme extract of *Mt*LPMO9B (lane 1) and *Mt*LPMO9C (lane 2). The pools of *Mt*LPMO9B (lane 4) or *Mt*LPMO9C (lane 5), used for various experiments, showed a single protein band with apparent molecular masses of 32 and 25 kDa, respectively (black arrows). The Precision Plus Protein (Bio-Rad Laboratories) was used as a marker. (lane 3 and 6). For more details about protein purification see Methods.

**Additional file 2: Figure S2.** LC/ESI–MS analysis of *Mt*LPMO9B and *Mt*LPMO9C. The purified a *Mt*LPMO9B- and b *Mt*LPMO9C-preparation was analyzed by LC/UV/ESI–MS using an AQUITY UPLC separation system and a SYNAPT ion mobilty mass spectrometer. The weighted average mass of *Mt*LPMO9B and *Mt*LPMO9C were 32,765 Da and 24,640 Da, respectively. ESI MS spectras (*m/z* values) of *Mt*LPMO9B and *Mt*LPMO9C show the presence of multiple glycations (+162 Da, hexose (180 Da) – water (18 Da)) of both LPMOs. Up to 13 and 5 glycosyl units are attached to *Mt*LPMO9B and *Mt*LPMO9C, respectively.

**Additional file 3: Figure S3.** MALDI-TOF mass spectrum of RAC incubated with *Mt*LPMO9B and *Mt*LPMO9C in the presence of ascorbic acid. a *Mt*LPMO9B incubated with RAC (RAC; 2 mg x g^−1^) in the presence of ascorbic acid. Clusters of C1-oxidized (GlcOS_n_^#^) and non-oxidized (GlcOS_n_) gluco-oligosaccharides were determined as their lithium (Li) adducts. Double Li-adducts are formed by exchanging a H^+^ ion for another Li^+^ ion (marked as GlcOS_n_^#^§). b *Mt*LPMO9C incubated with RAC (RAC; 2 mg x g^−1^) in the presence of ascorbic acid. Clusters of gluco-oligosaccharides oxidized at the C4 position (GlcOS_n_*) and non-oxidized gluco-oligosaccharides (GlcOS_n_) were determined as their lithium adducts. a and b Clusters of non-oxidized and oxidized gluco-oligosaccharides differ by a mass difference of one glucose unit (GlcOS_1_, 180 Da – 16 Da = 162 Da). See Fig. 1 for more details.

**Additional file 4: Figure S4.** HPAEC elution patterns of β-(1 → 3, 1 → 4)-glucan from oat spelt and xyloglucan incubated with *Mt*LPMO9C. Incubation of a oat spelt β-(1 → 3, 1 → 4)-glucan (2 mg x mL^−1^) and b xyloglucan from tamarind seed (XG; 2 mg x mL^−1^) with *Mt*LPMO9C (10 mg x g^−1^ substrate) with ascorbic acid (1 mM) or without. Samples were incubated in a 50 mM ammonium acetate (pH 5.0) for 24 h at 52 °C. a Numerous products (black arrows) were formed from oat spelt β-(1 → 3, 1 → 4)-glucan incubated with *Mt*LPMO9C in the presence of ascorbic acid compared to oat spelt β-(1 → 3, 1 → 4)-glucan without *Mt*LPMO9C addition in the presence of ascorbic acid. No oligosaccharides were released if oat spelt β-(1 → 3, 1 → 4)-glucan was incubated with *Mt*LPMO9C in the absence of ascorbic acid. b Incubation of XG with *Mt*LPMO9C in the presence of ascorbic acid released numerous products (black arrows) which were not present if XG was incubated with *Mt*LPMO9C in the absence of ascorbic acid. No oligosaccharides were formed from XG incubated with *Mt*LPMO9C in the absence of ascorbic acid.

**Additional file 5: Figure S5.** MALDI-TOF mass spectrum of β-(1 → 3, 1 → 4)-glucan from oat spelt incubated with *Mt*LPMO9C. a *Mt*LPMO9B incubated with oat spelt β-(1 → 3, 1 → 4)-glucan (2 mg x mL^−1^) in the presence of ascorbic acid. Clusters of C4-oxidized (GlcOS_n_^*^) and non-oxidized (GlcOS_n_) gluco-oligosaccharides were determined as their lithium (Li) adducts. Clusters of non-oxidized and C4-oxidized gluco-oligosaccharides differ by a mass difference of one glucose unit (GlcOS_1_, 180 Da – 16 Da = 162 Da). b (enlargement of a) Several additional peaks were determined showing the characteristic 2 Da lower mass as reported for C4-oxidized products (GlcOS_n_* = GlcOS_n_ – 2 Da). See Methods for more details.

**Additional file 6: Figure S6.** MALDI-TOF mass spectrum of xyloglucan incubated with *Mt*LPMO9C. a *Mt*LPMO9C incubated with xyloglucan (2 mg x mL^−1^) in the presence of ascorbic acid. Xyloglucan oligosaccharide (XG-OS) clusters of C4-oxidized (XG-OS_n_^*^) and non-oxidized (XG-OS_n_) oligosaccharides were determined (*m*/*z* values) as their lithium (Li) adducts. b (enlargement of a) Several additional peaks were annotated showing the characteristic 2 Da lower mass as reported for C4-oxidized products (GlcOS_n_* = GlcOS_n_ – 2 Da). An identification of non-oxidized and C4-oxidized gluco-oligosaccharides of different substituted xyloglucan oligosaccharides based on Fry et al. [49] remains limited due to the low amounts of products released from xyloglucan incubated with *Mt*LPMO9C (Additional file 4) and the therefore impossible MS^2^-fragmentation [49]. Compounds annotated as GlcOS_n_XOS_n_ indicate the number of expected hexoses and pentoses to be present in xyloglucan oligosaccharides. Samples were incubated in 50 mM ammonium acetate buffer (pH 5.0) containing 1 mM ascorbic acid for 24 h at 52 °C. See Methods for more details.

**Additional file 7: Figure S7.** Cartoons of the highly conserved surface patch near the Gly-Pro-Gly triad. Surface charge distribution and enlargement (in brackets) of a *Mt*LPMO9A (d), b *Mt*LPMO9B (e) and c *Mt*LPMO9C (f) emphasize the highly conserved surface patch near the Gly-Pro-Gly triad, positioned in the amino acid sequence around residue number 200 [21, 29]. The scaling from the negative and positive electrostatic potential regions are -5 for blue and +5 for the red regions. The electrostatic map was obtained from APBS plugin from PyMOL. Protein orientation: the flat substrate-binding site is located at the bottom of all three LPMOs indicated by the black arrow.

## Abbreviations

AA: auxiliary activity
AEC: anion-exchange chromatography
AUC: area under the curve
BiWX: birchwood glucuronoxylan
CAZy: carbohydrate-active enzymes
CBM: carbohydrate-binding module
CDH: cellobiose dehydrogenase
GH: glycoside hydrolases
GlcAmeXOS_n_: 4-*O*-methylglucoronic acid containing xylo-oligosaccharides
GlcOS_n_: gluco-oligosaccharides
^#^: C1 oxidized
*: C4 oxidized
HPAEC: high-performance anion-exchange chromatography
LC/ESI-MS: liquid chromatography/electrospray ionization-mass spectrometry
LPMO: lytic polysaccharide monooxygenase
MALDI-TOF MS: matrix-assisted laser desorption ionization–time of flight mass spectrometry
OSX: oat spelt xylan
PAD: pulsed amperometric detection
PMO: polysaccharide monooxygenase
RAC: regenerated amorphous cellulose
SEC: size exclusion chromatography
SDS-PAGE: sodium dodecyl sulfate-polyacrylamide gel electrophoresis
WAX: wheat arabinoxylan
