# Supplementary figures and images for: Lytic polysaccharide monooxygenases from Myceliophthora thermophila C1 differ in substrate preference and reducing agent specificity
Source: Biotechnol Biofuels. 2016 Aug 31;9:186. doi: 10.1186/s13068-016-0594-y (PMC5007705; doi:10.1186/s13068-016-0594-y)

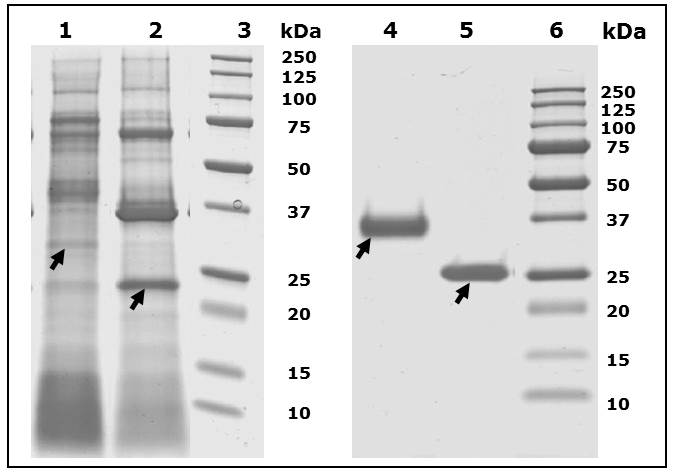

Supplement: Supplementary file 1 — Additional file 1: Figure S1. SDS-PAGE of MtLPMO9B and MtLPMO9C fractions during enzyme purification. MtLPMO9B and MtLPMO9C were purified by multiple chromatographic steps from the crude enzyme extract of MtLPMO9B (lane 1) and MtLPMO9C (lane 2). The pools of MtLPMO9B (lane 4) or MtLPMO9C (lane 5), used for various experiments, showed a single protein band with apparent molecular masses of 32 and 25 kDa, respectively (black arrows). The Precision Plus Protein (Bio-Rad Laboratories) was used as a marker. (lane 3 and 6). For more details about protein purification see Methods. [file 13068_2016_594_MOESM1_ESM.jpg]

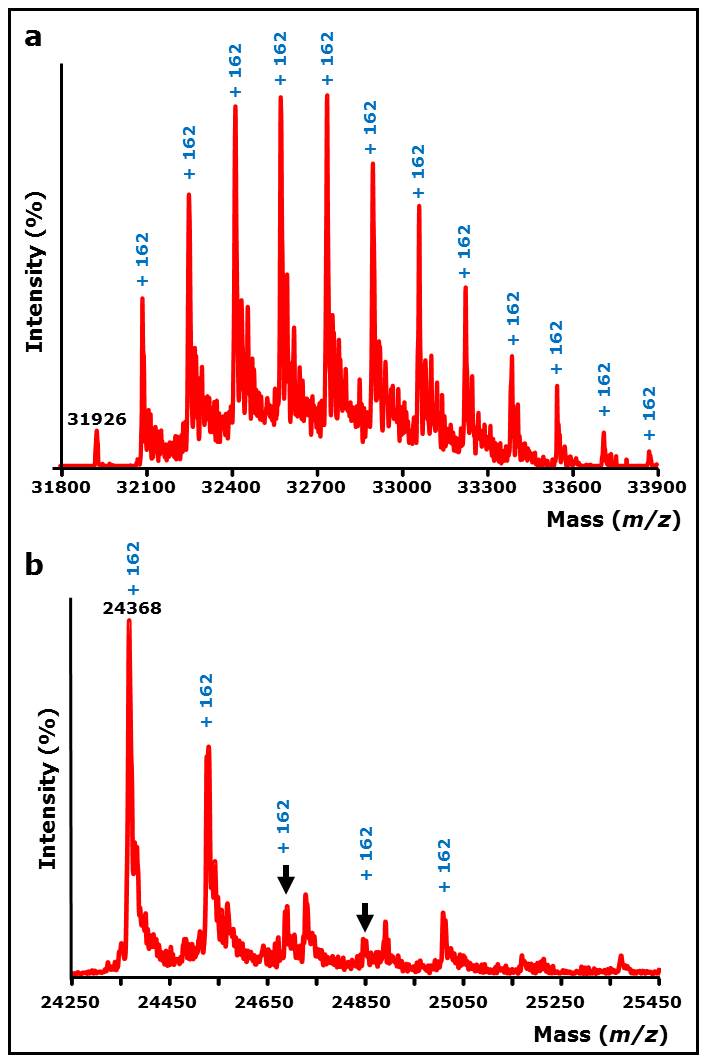

Supplement: Supplementary file 2 — Additional file 2: Figure S2. LC/ESI–MS analysis of MtLPMO9B and MtLPMO9C. The purified a MtLPMO9B- and b MtLPMO9C-preparation was analyzed by LC/UV/ESI–MS using an AQUITY UPLC separation system and a SYNAPT ion mobilty mass spectrometer. The weighted average mass of MtLPMO9B and MtLPMO9C were 32,765 Da and 24,640 Da, respectively. ESI MS spectras (m/z values) of MtLPMO9B and MtLPMO9C show the presence of multiple glycations (+162 Da, hexose (180 Da) – water (18 Da)) of both LPMOs. Up to 13 and 5 glycosyl units are attached to MtLPMO9B and MtLPMO9C, respectively. [file 13068_2016_594_MOESM2_ESM.jpg]

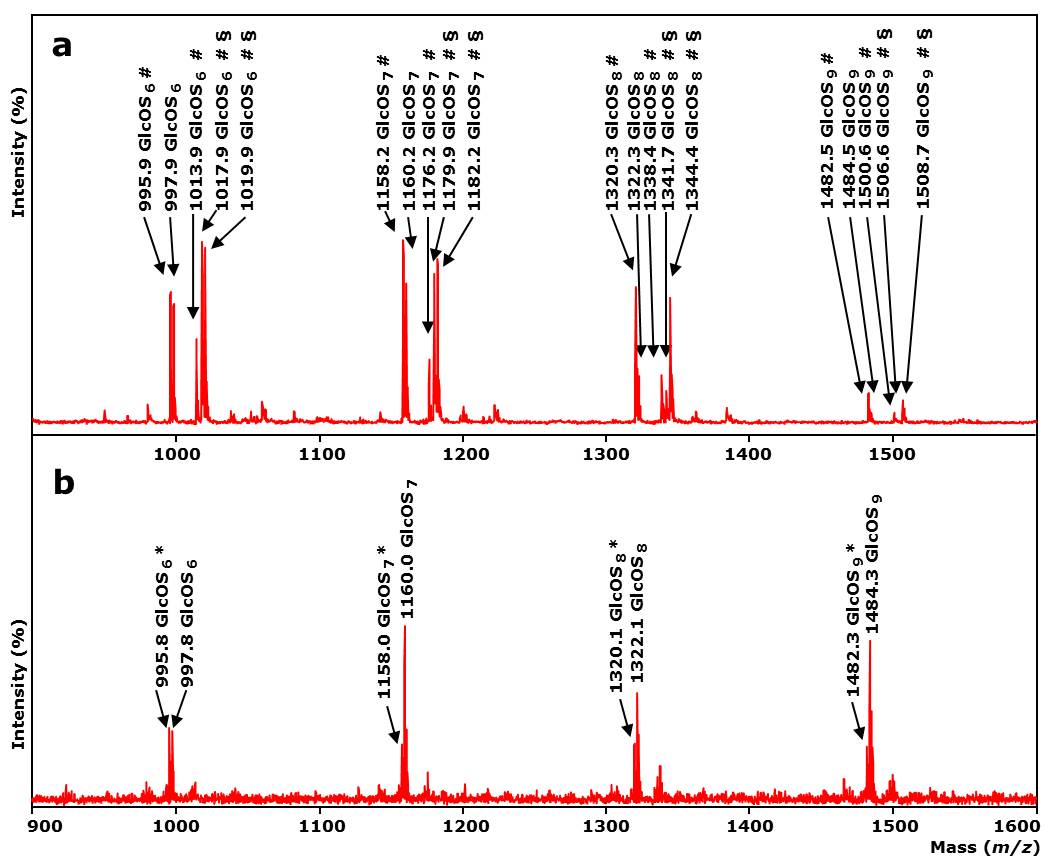

Supplement: Supplementary file 3 — Additional file 3: Figure S3. MALDI-TOF mass spectrum of RAC incubated with MtLPMO9B and MtLPMO9C in the presence of ascorbic acid. a MtLPMO9B incubated with RAC (RAC; 2 mg x g−1) in the presence of ascorbic acid. Clusters of C1-oxidized (GlcOSn#) and non-oxidized (GlcOSn) gluco-oligosaccharides were determined as their lithium (Li) adducts. Double Li-adducts are formed by exchanging a H+ ion for another Li+ ion (marked as GlcOSn#§). b MtLPMO9C incubated with RAC (RAC; 2 mg x g−1) in the presence of ascorbic acid. Clusters of gluco-oligosaccharides oxidized at the C4 position (GlcOSn*) and non-oxidized gluco-oligosaccharides (GlcOSn) were determined as their lithium adducts. a and b Clusters of non-oxidized and oxidized gluco-oligosaccharides differ by a mass difference of one glucose unit (GlcOS1, 180 Da – 16 Da = 162 Da). See Fig. 1 for more details. [file 13068_2016_594_MOESM3_ESM.jpg]

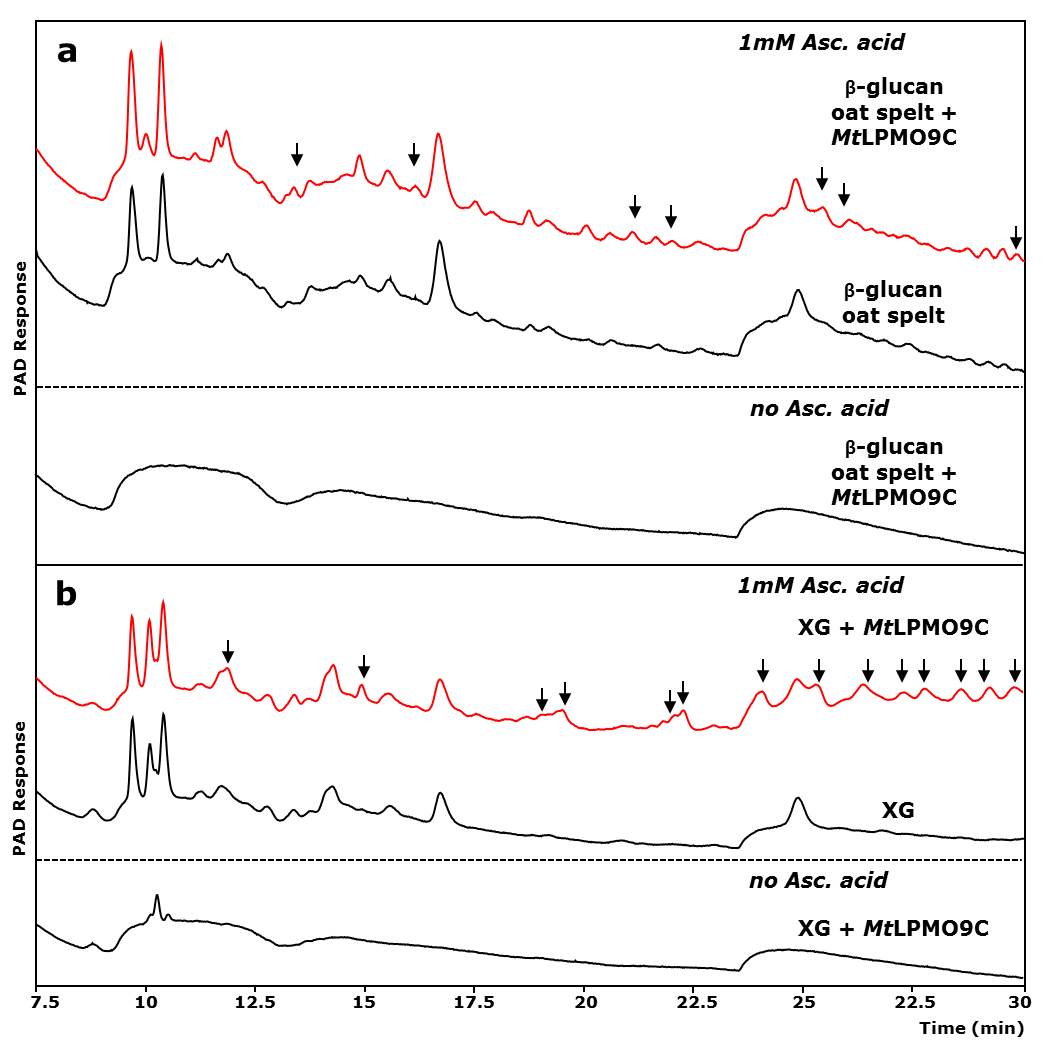

Supplement: Supplementary file 4 — Additional file 4: Figure S4. HPAEC elution patterns of β-(1 → 3, 1 → 4)-glucan from oat spelt and xyloglucan incubated with MtLPMO9C. Incubation of a oat spelt β-(1 → 3, 1 → 4)-glucan (2 mg x mL−1) and b xyloglucan from tamarind seed (XG; 2 mg x mL−1) with MtLPMO9C (10 mg x g−1 substrate) with ascorbic acid (1 mM) or without. Samples were incubated in a 50 mM ammonium acetate (pH 5.0) for 24 h at 52 °C. a Numerous products (black arrows) were formed from oat spelt β-(1 → 3, 1 → 4)-glucan incubated with MtLPMO9C in the presence of ascorbic acid compared to oat spelt β-(1 → 3, 1 → 4)-glucan without MtLPMO9C addition in the presence of ascorbic acid. No oligosaccharides were released if oat spelt β-(1 → 3, 1 → 4)-glucan was incubated with MtLPMO9C in the absence of ascorbic acid. b Incubation of XG with MtLPMO9C in the presence of ascorbic acid released numerous products (black arrows) which were not present if XG was incubated with MtLPMO9C in the absence of ascorbic acid. No oligosaccharides were formed from XG incubated with MtLPMO9C in the absence of ascorbic acid. [file 13068_2016_594_MOESM4_ESM.jpg]

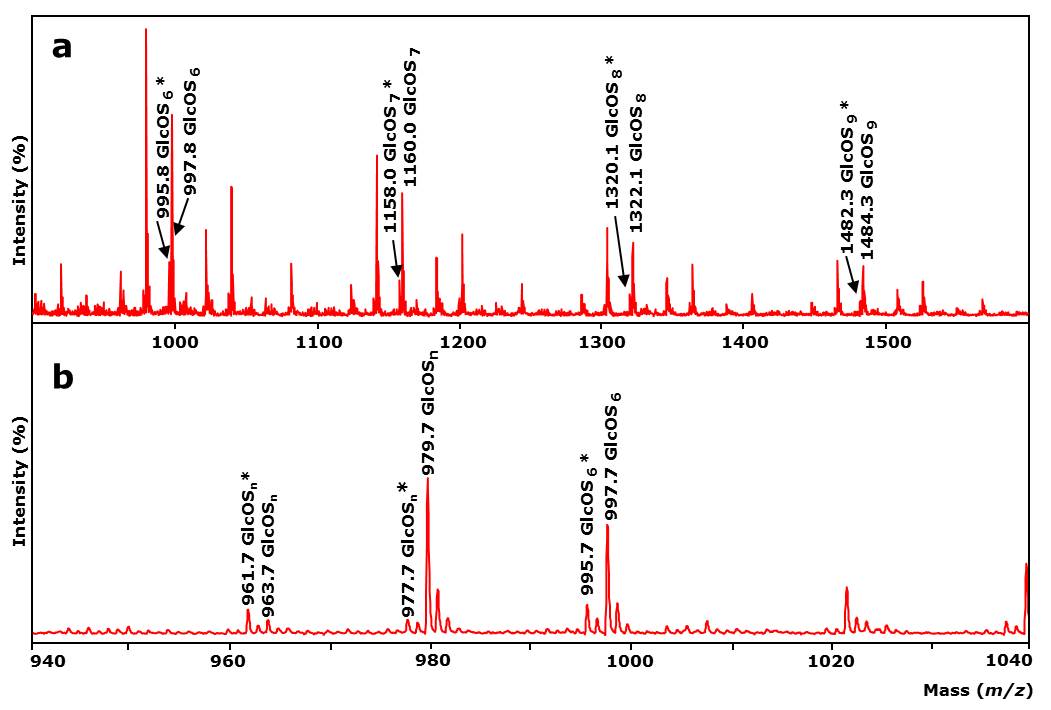

Supplement: Supplementary file 5 — Additional file 5: Figure S5. MALDI-TOF mass spectrum of β-(1 → 3, 1 → 4)-glucan from oat spelt incubated with MtLPMO9C. a MtLPMO9B incubated with oat spelt β-(1 → 3, 1 → 4)-glucan (2 mg x mL−1) in the presence of ascorbic acid. Clusters of C4-oxidized (GlcOSn*) and non-oxidized (GlcOSn) gluco-oligosaccharides were determined as their lithium (Li) adducts. Clusters of non-oxidized and C4-oxidized gluco-oligosaccharides differ by a mass difference of one glucose unit (GlcOS1, 180 Da – 16 Da = 162 Da). b (enlargement of a) Several additional peaks were determined showing the characteristic 2 Da lower mass as reported for C4-oxidized products (GlcOSn* = GlcOSn – 2 Da). See Methods for more details. [file 13068_2016_594_MOESM5_ESM.jpg]

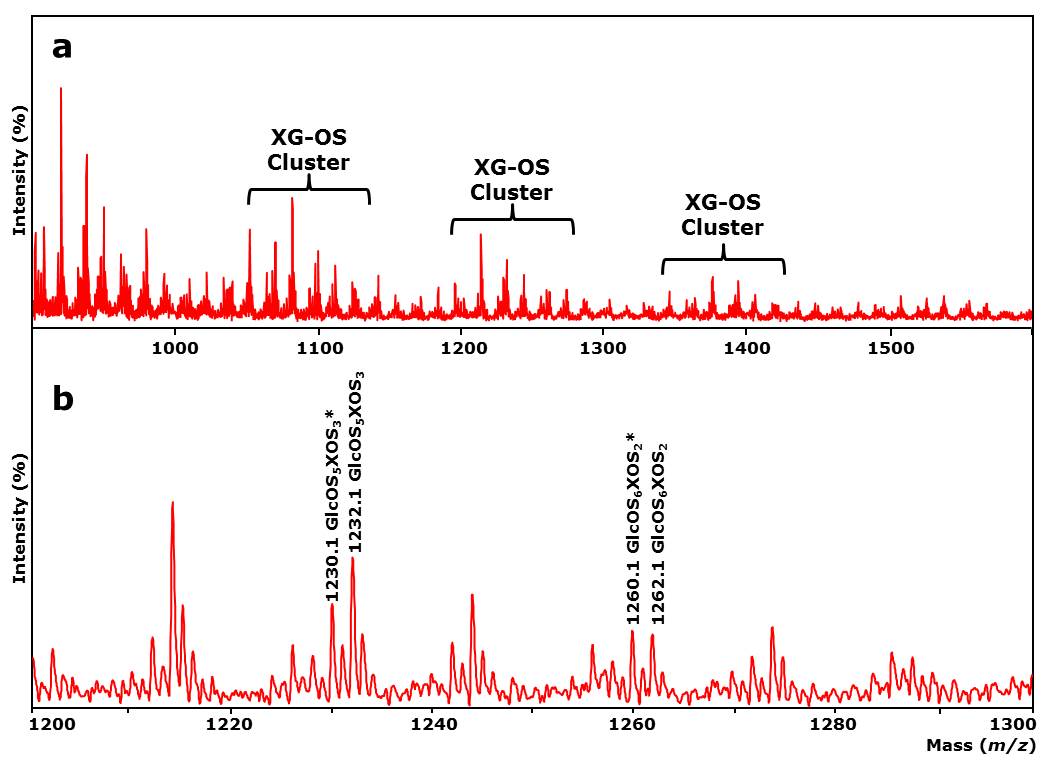

Supplement: Supplementary file 6 — Additional file 6: Figure S6. MALDI-TOF mass spectrum of xyloglucan incubated with MtLPMO9C. a MtLPMO9C incubated with xyloglucan (2 mg x mL−1) in the presence of ascorbic acid. Xyloglucan oligosaccharide (XG-OS) clusters of C4-oxidized (XG-OSn*) and non-oxidized (XG-OSn) oligosaccharides were determined (m/z values) as their lithium (Li) adducts. b (enlargement of a) Several additional peaks were annotated showing the characteristic 2 Da lower mass as reported for C4-oxidized products (GlcOSn* = GlcOSn – 2 Da). An identification of non-oxidized and C4-oxidized gluco-oligosaccharides of different substituted xyloglucan oligosaccharides based on Fry et al. [49] remains limited due to the low amounts of products released from xyloglucan incubated with MtLPMO9C (Additional file 4) and the therefore impossible MS2-fragmentation [49]. Compounds annotated as GlcOSnXOSn indicate the number of expected hexoses and pentoses to be present in xyloglucan oligosaccharides. Samples were incubated in 50 mM ammonium acetate buffer (pH 5.0) containing 1 mM ascorbic acid for 24 h at 52 °C. See Methods for more details. [file 13068_2016_594_MOESM6_ESM.jpg]

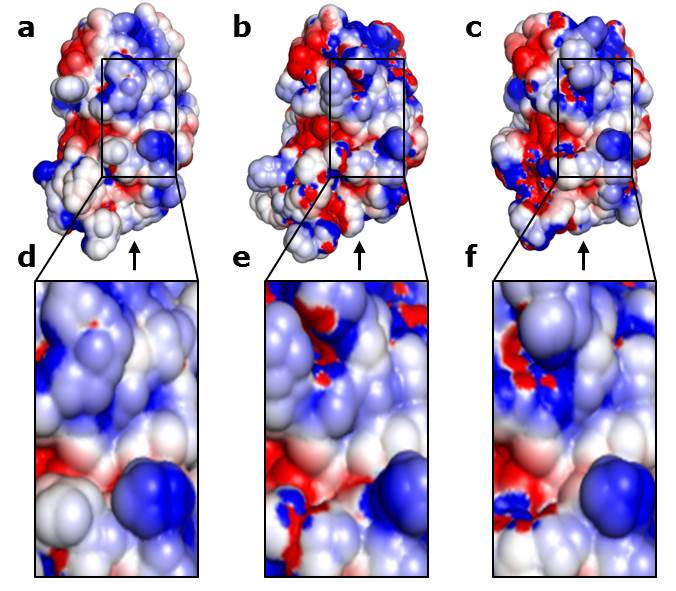

Supplement: Supplementary file 7 — Additional file 7: Figure S7. Cartoons of the highly conserved surface patch near the Gly-Pro-Gly triad. Surface charge distribution and enlargement (in brackets) of a MtLPMO9A (d), b MtLPMO9B (e) and c MtLPMO9C (f) emphasize the highly conserved surface patch near the Gly-Pro-Gly triad, positioned in the amino acid sequence around residue number 200 [21, 29]. The scaling from the negative and positive electrostatic potential regions are -5 for blue and +5 for the red regions. The electrostatic map was obtained from APBS plugin from PyMOL. Protein orientation: the flat substrate-binding site is located at the bottom of all three LPMOs indicated by the black arrow. [file 13068_2016_594_MOESM7_ESM.jpg]
